# Editorial

**Published:** 1863-01

**Authors:** 


### Editorial.

### AMERICAN MEDICAL ASSOCIATION.

Office, Medical Examiner,
Chicago, *February 20th*, 1863.

The next regular Annual Meeting of the American Medical Association will be held in the City of Chicago, Illinois, on the first Tuesday in June, 1863. Every permanently organized State, County, and Local Medical Society is entitled to send one Delegate for every ten members, and one additional Delegate for a fraction of more than half of that number. Medical Colleges, and Hospitals containing over 100 beds for the sick, are entitled to two Delegates; and all other permanently organized Medical Institutions are entitled to one Delegate each.

The Committee earnestly desire a full attendance from all parts of the country.

By order of the Committee of Arrangements,
N. S. DAVIS, *Chairman.*

We call the earnest attention of our readers to the above notice of the Annual Meeting of the American Medical Association. For two years past, the Committee of Arrangements have been constrained by the almost unanimous advice of members in all sections of the country, to postpone the Annual Meetings. Recently, however, one of the oldest and most influential medical organizations in the country: the New York State Medical Society, has unanimously recommended a meeting at the regular time, the present year. The same advice has been received from other quarters; and, though some still advise a further postponement, the Committee, anxious only to comply with the wishes of the profession, and to sustain the national organization, have deemed it advisable to issue the usual notice for a regular meeting.

Every necessary arrangement has been made to accommodate the meetings, both of the Association in General Assembly, and in Sections; and we can assure the profession everywhere, that they will be as cordially welcomed in Chicago, as they have been in any city heretofore visited by the Association.

### ILLINOIS STATE MEDICAL SOCIETY.

The regular Annual Meeting of the Illinois State Medical Society will be held at Jacksonville, on the *first* Tuesday in May next; commencing at 10 o'clock in the morning. We hope the profession in every part of the State will be represented, as the meeting will be an important and interesting one.

No further postponement will be made on account of the continuance of the rebellion.

N. S. DAVIS,

*Permanent Sec'y Ill. State Med. Society.*

Chicago, *Feb.* 13*th*, 1863.

We trust the above notice will receive due attention from all our readers in this State.

We are assured that the Committee of Arrangements will do all they can to make the meeting a very pleasant, as well as a profitable one. Every County and Local Society should see that such delegates are appointed as will attend; and members of the profession in cities and counties where no social organization exists, may rely on being made members, either permanently or temporarily, by invitation, if they are present at the meeting. Let all who desire to maintain our State organization, aid in extending the notice of the meeting among their professional friends and acquaintances.

STATE OF ILLINOIS,
OFFICE OF THE BOARD OF MEDICAL EXAMINERS,
CHICAGO, *March* 9, 1863.

DR. N. S. DAVIS,
EDITOR OF MEDICAL EXAMINER,

SIR:—Many of your readers will be interested to know the following, in regard to the Military Medical Service:—

There are vacancies in the Illinois Regiments, in the places of Surgeons and Assistant-Surgeons, especially the latter.

Candidates first obtain an order from the Governor, or Adjutant-General, for examination; and, after being approved, apply to the Governor for a Commission.

The Examiners require satisfactory evidence of "professional standing, moral character, and sobriety."

The examinations embrace all the departments of Medicine and Surgery.

Respectfully,
HENRY WING, M.D.,
*Sec'y of Board of Examiners.*

---

NEW BOOKS.—We have received several valuable works, such as, "Clinical Lectures on Diseases of Women," by J. Y. SIMPSON; a new edition of Bedford's Obstetrics; Transactions of the New York Academy of Medicine, and a variety of pamphlets. The unexpected length of the article of Prof. ANDREWS, on the Surgical results of the Battle of Vicksburg, leaves us no space for the usual Book Notices in the present number. We shall endeavor to do them justice in our next issue.

---

COMMUNICATIONS.—Interesting papers have been received from Drs. JONES and TREAT of Janesville, Wis., and from Prof. BYFORD of this city. They will be found in the next number of the *Examiner*.

In the next number, we shall also resume the publication of regular Clinical Reports of what is doing in the Hospitals and Dispensaries of this city.

Artificial Limbs.—Hereafter, soldiers entitled to artificial limbs, and not in one of the U. S. Hospitals established for their reception, may, upon presenting proper proof to any of the following duly appointed Medical directors, receive from them an order for the same.

*Names of Medical Directors.*—Surgeons A. N. McLaren, U. S. A., Boston, Mass.; Chas. McDougall, U. S. A., New York, N. Y.; W. S King, U. S. A., Philadelphia, Pa.; I. Simpson, U. S. A., Baltimore. Md.; R. O. Abbott, U. S. A., Washington, D. C.; L. H. Holden, U. S. A., Cincinnati, O.; J. F. Head, U. S. A., Louisville, Ky.; M. Mills, U. S. A., St. Louis, Mo.; I. B. Potter, U. S. A., Chicago, Ill.; R. H. Alexander, U. S. A., New Orleans, La.

These orders may be given as desired in each individual case, upon any of the following manufacturers: Palmer, Selpho, Bly, Hudson, or Jewett, and the price of the limb furnished by these dealers on such orders is not to exceed fifty dollars.—*Med. and Surg. Reporter.*

---

Medical Qualifications.—Mr. Postgate, in his very able introductory lecture at Birmingham, thus sums up the requisite qualifications for the study of medicine:—1. Good health, without which, all thoughts and all efforts are puny, incomplete, and inoperative. 2. A well-balanced and an evenly regulated mind. 3. Unselfishness. 4. Fixity of purpose. 5. An unswerving determination to do always what is right, let the consequences be what they may. 6. Clearness of perception. 7. Promptness of action. 8. General benevolence; and, I will add, 9. General contempt for the luxuries and comforts of life, looking for reward to that satisfaction, peace and contentment of conscience, which flows from the conviction of human misery alleviated, and of human life prolonged, by duties faithfully discharged and services cheerfully rendered.—*Dublin Medical Press.*

---

### CIRCULAR TO PHYSICIANS.

Surgeon-General's Office,
Washington, *February* 20, 1863.

The Surgeon-General would remind the medical profession that, some months since, a medical officer was detailed by the Department, to prepare the surgical history of the rebellion. It is intended that this history shall embrace, among other topics, the collected results of the gun-shot injuries of the war, and of the operations performed for their relief.

Many facts bearing on these subjects can be obtained by an examination of the returns of the various military hospitals; and explicit orders have been issued to the surgeons in charge, as to the manner of reporting. Yet it is found, practically, that the results of all cases cannot be included in these reports.

In every depot of wounded, and after every action, there exists a large class of injured men, who, in various stages of convalescence, pass from the observation and treatment of the military surgeon, and are lost sight of by the medical department. These patients are those who are either furloughed, or discharged the service by military authority, before their treatment is entirely terminated. Under such circumstances, all past records of these cases are rendered valueless from the absence of a positive knowledge of their results.

To remedy this evil, the Surgeon-General appeals to the profession of the country, and solicits their co-operation. He would ask every physician and surgeon who may be called upon to treat any officer or soldier, wounded in the service, carefully to note the results of the case, to record his observations, and when the case shall have terminated, to transmit a copy of his observations to the Surgeon-General's office.

The following form is suggested:—

FORM.

Date of Communication.

Character of Injury. Name and address of Physician forwarding it.

| | Where wounded and Date. | To what Hospital Transported. | What operation, etc., Performed. | By whom Performed. | Date of Furlo'gh or Discharge. | Present condition of Patient. Account of case, Treatment, etc. Result. |
|---|---|---|---|---|---|---|
| Patient's Name and Age, | | | | | | |
| " Rank, | | | | | | |
| " Reg't & Co'y, | | | | | | |
| " Post Address, | | | | | | |

In all cases of recovery after *excisions* of bone the amount and character of the movements executed by the patient with the injured limb, should be accurately described. Where amputation has been practiced, the character of the stump should be noted, especially when the operation has been performed through an articulation. In cases of compound fracture, the point of fracture should be stated, as also the degree of efficiency of the limb remaining after treatment. In compound fractures of the femur, the amount of shortening should be measured, and the strength and usefulness of the limb described. In those patients in whom injuries of the skull have occurred, or upon whom the trephine has been applied, the mental and physical conditions should alike be dwelt upon.

In thus placing before the profession the object he desires to obtain, the Surgeon-General trusts that he will meet with active co-operation. By the means above indicated, much information that is valuable may be collected, and the interests of the science of surgery materially advanced.

W. A. HAMMOND,
*Surgeon-General, U.S.A.*

—*American Medical Times.*

### USE OF TOBACCO.

Sir Ranald Martin expresses, in his recent work on *Tropical Climates*, the following opinion of the use of tobacco :—

"There is another habit respecting which I shall venture to say a few words, because it is both a bad one and a comparatively new one—I mean the immoderate use of Tobacco—a habit brought amongst us from the continent of Europe, on the cessation of the French revolutionary war. Young military men are apt to regard the habit as a manly one, until severe dyspepsia, giddiness, shattered nerves, sallow complexion, disturbed action of the heart, and other symptoms show themselves, and then it is frequently too late to stop. 'The sallow complexions, black, broken, and unsound teeth' of the Germans are matters of notoriety to all travellers. 'You may,' says one of them, 'smell a German in any part of the room, or scent him at a quarter of a mile's distance in the open air, if the wind be favorable.'

"Much is talked of the good effects of tobacco-smoking in damp and malarious localities, by persons who, in defiance of geographical differences, carry the habit wherever they go—from the marshes of Burmah to the arid plains of Hindustan, forgetting that, meanwhile, in the language of Cassio, 'they put an enemy in their mouths to steal away their brains;' but I think there is good reason to question the benefits of this habit of smoking even in the fatherland of fog and damp, or that tobacco ever acts as a preventive to any disease, and least of all to fever.

"The truth is, that many persons puff themselves into the good graces of snobs and spoonies like themselves, and use cigars by the score now, as Lord Chesterfield drank and smoked in his time, notwithstanding his aversion to wine and tobacco—'because he thought such practices very *genteel*, and made him look like a man.' How his lordship may have looked under the united influence of wine and tobacco, his biographers have failed to relate; but we all know how our modern 'spoonies' and 'snobs' in our thoroughfares look, after a course of cigar-smoking alone."—*Med. News and Lib.*

---

Reported Killed.—Dr. Thos. N. Penrose, of this city, Assistant-Surgeon on board the Harriet Lane, is reported to have been killed in recent capture of that vessel at Galveston, Texas, by the rebels.—*Med. and Surg. Reporter.*

